# Notes and News

**Published:** 1889-09-07

**Authors:** 


### NOTES AND NEWS.

COLLECTED AND COMMUNICATED.

*Chambers' Journal* for September contains an account of a visit to Miss Derham's Home for Semi-convalescent Children at Bushey Heath. Children suffering from abscesses, open wound, etc., are taken there and tended for seven shillings a week.

AUSTRALIA has only a partial belief in M. Pasteur. It does not regard him as the saviour of its crops from the rabbit pest; but the farmers are greatly pleased with some experiments undertaken by the agents of the great chemist in vaccinating sheep to protect them from anthrax. Much loss is caused to the farmers by this disease, and M. Pasteur has been asked to appoint an agent who would vaccinate the sheep at fixed charges.

SOME shocking instances of drunkenness amongst children in Austria are being quoted in the daily papers. A child of four and another of eight died a short time ago from the effects of drink. Four children became the victims of epileptic fits from the same cause. One child—a boy of five—took two or three glasses of brandy a day; a boy of four was accustomed to drink sixty to eighty grammes of cognac. In a large number of cases of nervous affections the origin has been traced to strong liquor. The thought of a child suffering from delirium tremens is too horrible to contemplate.

THE Anthropological Congress at Paris has had a big question before it. The problem propounded for discussion was the mental and physical differentiation between habitual criminals and persons of good morals and exemplary conduct. Dr. Lombroso, the famous specialist in criminality from Turin, was of opinion that there existed a distinct type of criminal man, just as a savage type is still to be met with. From his post-mortem examinations of the bodies of criminals he found that all the subjects presented the same characteristics—wide eye-orbits, prominent cheekbones, distended nostrils, and other anatomical appearances which were also peculiar to savages. Crime, in Dr. Lombroso's opinion, was also the result of atavism to a great extent. Other Italian physicians corroborated these observations. M. Manouvrier, of Paris, argued that there were no characteristics by which criminals as a class or type could be distinguished from other men. It was, of course, true that certain kinds of anatomical characterisation were found most frequently among criminals; but he objected to too much generalisation in the matter. A Lyons doctor, in a report on the influence of the professions on crime, stated that the largest percentage of malefactors who belonged to the better classes was among agricultural and trading people.

AFTER the formal ceremony of opening the Victoria Eye Infirmary, Hereford, a series of revels and a sale of work was held in the grounds. A very pretty maypole dance had been arranged by the Rev. H. Gascoigne, and in the evening a concert was held in the large marquee. The whole town seemed bent on keeping holiday, and at night there were brilliant illuminations and fireworks. Altogether £340 was realised, including £56 taken at the gates, £30 from the sale of work, about £5 at the concert.

DIPHTHERIA is on the increase throughout England, and quite a scare has been caused at East Haddon, in Northamptonshire, by its sudden and fatal appearance there. There are cases in half the dwellings in the parish, and five deaths have occurred in two days. A house has been turned into a temporary infirmary, and nurses have been sent by the local authority. The inhabitants are described as almost panic-stricken. The sanitary condition of the place is very bad, and it is feared that the epidemic will increase.

THE New Hospital for Women Committee are already starting their friends to work for their April bazaar. It is contemplated to invite the Princess of Wales to open the bazaar. A bazaar committee is being formed, and those with experience in the organisation of bazaars on a large scale are invited to send their names to the secretary, 222, Marylebone-road, N.W., and to volunteer either to hold stalls or provide goods. The committee suggest that stalls should be held for the sale of the following groups of articles: Children's clothing, ladies' and household lingerie, gift clothing for the poor, fine art; Indian, Chinese, and Japanese goods; books, baskets, pottery of all nations, fruit and flowers, refreshments, live stock, and miscellaneous.

STOCKTON HOSPITAL sends a good report forth to the public. During the last year 473 cases have been treated for an average of 33½ days each, this being in addition to 1,265 outdoor patients, and making altogether 1,738. The whole of the forty beds have, for the first time, been occupied during the past year, and by the new wing now in course of construction, greatly increased facilities are being provided, and an extended sphere of usefulness may be anticipated. The subscriptions in 1888-9 have amounted to £1,609 3s. 9d., being in excess of any former year, as also have been annual subscriptions and donations. The special fund for the new wing is progressing, and it is hoped it will shortly been opened free of debt.

THE Victoria Eye and Ear Hospital, Hereford, was opened in August by Lady Bailey, the wife of the member. The building is handsome and well situated. The ruling feature of the internal arrangement is a central corridor, 7ft. wide, which is parallel with the street, having the rooms on the right and left. This system is undoubtedly the most economical for the site. Another peculiar feature of the plan is the cross ventilation of the wards by windows. In connection with the ventilation, care is also taken to place intercepting lobbies between the rooms and usual offices. In the front of the building, on the ground floor, are the waiting-room, secretary's office, matron's dayrooms, and general dining-room, the first and last having bay windows. There are four wards, one larger and one smaller ward for each sex, at either wing of the building. The two larger wards, for the present, will only have four beds each, and a child's cot or two; and the smaller ones, two beds each. Each large ward has a nurse's room adjoining, and by means of a "duty window" the nurse see into the wards at any hour. Mr. Lingen Barber is the architect. The friendly societies of the town have furnished one of the wards.

The following vacancies are announced this week, the amount of the salary in each case being given after the name of the institution:

**Resident Medical Officers.**

Victoria Park Hospital for Diseases of the Chest. House Physician.—Board, &c.
Wolverhampton Hospital. House Physician.—£100, board, &c.
Stamford Infirmary. House Surgeon and Secretary.—£100, board, &c.
Royal Albert Hospital, Devonport. Medical Officer.—£150, board, &c.
North London Consumption Hospital. Medical Officer.—£40, board, &c.
Poplar Hospital. Assistant House Surgeon.—£50.
Macclesfield Infirmary. Senior House Surgeon.—£100, board, &c.
Shrewsbury County Asylum. Junior Medical Officer.—£100, board, &c.

**Non-Resident Medical Officers.**

Glasgow Royal Infirmary. Curator of Museum and Pathologist.—£50.
Aberdeen University. Six Examiners in Medicine.—£30.
Manchester Royal Infirmary. Honorary Assistant Surgeon.

---

On Saturday, the 31st ult., at the City Hospital, South Grafton-street, Liverpool, a farewell supper was given by the matron on the occasion of Nurse Symes resigning the post of charge nurse (which she has held for three years) to be married. After supper a very handsome tea service was presented by the matron and charge nurses. The remainder of the evening was very pleasantly spent.

---

The population question still troubles France, and still from the anti-Malthusian point of view. The excess of births over deaths, which was 108,000 in 1881, has steadily sunk till it is now only 44,000. In 43 departments, indeed, out of 87 there is an excess of deaths over births. The census for the first time contains details on the foreign element, some of which should shame this country. Of 475 English births, 77 or 16 per cent. were illegitimate, but, taking Paris alone, 38 per cent. of the English births were illegitimate. The percentage of illegitimacy was 24 among the Germans (but 33 in Paris), 17 among the Swiss (21 in Paris), 13 among the Belgians, 11 among the Italians, and 7 among the Spaniards.

---

A quarterly general court of the governors of the Middlesex Hospital was held on Thursday, 29th August, the Right Hon. Lord Sandhurst taking the chair. Among those present were Mr. J. G. Noel, C.B., Mr. Larkins, J.P., Mr. Francis Hoare, Lieut.-Col. Holloway, Dr. Cayley, Dr. Henry Thompson, Dr. Beecher, and the Rev. Theophilus Echalaz. Lord Sandhurst, chairman of the weekly board, in explaining the various matters mentioned in the minutes, alluded to the favourable prospects of the hospital, consequent upon the munificent bequest of Miss Mary Eason, which, in addition to the amount to be received from the estate of the late Mr. John Frederick Pike Scrivener, would swell the funds of the hospital in a very welcome manner.

---

The quarterly report of Belfast Royal Hospital has no better news financially than usual. At the close of last quarter's report the hospital stood indebted the sum of £1,463 18s. 8d. Since then the receipts have been £1,886 12s. 11d., and the expenditure £2,068 4s. 11d., which brings up the indebtedness to the sum of £1,645 10s. 8d. at the end of July. We must profess our belief that there is no necessity for such a large debt if systematic measures were taken to remove it. During the quarter there were treated in hospital 517 intern patients; of these 201 were medical, and 316 surgical; 83 operations were performed. During the same period there were treated as extern patients 5,208.

Just outside Berlin is a huge shelter for the destitute poor, which is steadily enlarging the scope of its labours. Since January 21 last there has been a provision of sick-rooms, with 100 beds, on the first floor of the main building. If any of the inmates should have sore feet, be feverish, exhausted from fatigue, or be otherwise not in a fit condition to start again on their travels, they may see the doctor, who, if he thinks fit, orders them into the sick-rooms. There they will be looked after for several days, at the end of which time, should they still be unable to leave owing to the gravity of their ailment, they are sent to the hospital. The staff engaged in the management of the shelter comprises a chief inspector, a "house father," twelve warders, and two clerks.

---

There was a very Scotch flavour about the bazaar at Broadford, Skye, in aid of the Mackinnon Memorial Hospital Fund. To begin with, the weather was "a wee bit saft," and most of the visitors arrived in yachts, and looked blown about and rosy, in their rough rain-proof dress. Then Ronald Mackenzie, of the Seaforth Highlanders, skirled forth martial airs from the pipes, also strathpeys, pibrochs, and reels. The sad necessity for a hospital in Skye arises from the prevalence of typhoid and famine fever, which are due to poverty and the insanitary condition of the crofters' cottages. The "stalwart Highlander" of the story books is in reality often sick and sorry, but he is also very independent, and we doubt if he will ever consent to become a hospital patient, unless he is at his last gasp.

---

The Cripplegate Charities are to be dispensed according to a new scheme. The trust, which will be known as the Cripplegate Foundation, is to be controlled by a governing body consisting of fifteen gentlemen, of whom three will be *ex officio* members—viz., the vicar and churchwardens for the time being of the parish. Subject to grants to the vicar sufficient to provide for the ecclesiastical requirements of the parish, the whole income of the trust is to be devoted to two branches of work—eleemosynary and institute. The governing body may also provide, by means of nursing dispensaries, hospitals, convalescent homes, emigration grants, or otherwise, medical and other relief to poor inhabitants of the parish. The remainder of the income of the foundation is to be devoted to the purchase of a building to be known as the Cripplegate Institute, which the Commissioners direct shall be used as a library, reading-room, etc.

The story of St. Leonards Hospital, Sudbury, formed the theme of a late address by Dr. Holden to a branch of the British Medical Association, and now it issues its twenty-first report. The hospital is prettily situated, and has separate grounds for the use of male and female patients. It is built in the form of two pavilion wards, one on each side of a two-storeyed tower. This central part contains below two separation wards, matron's room, surgery, and operating-room, and above bedrooms and kitchen. It is a great advantage in a small hospital to have the kitchen in the upper part of the building, and well away from the wards. There were 123 patients admitted during last year, of whom sixty-two were males and sixty-one females. Seventy-three patients were discharged cured, twenty-nine were relieved, eight were unrelieved, two were sent to the Addenbrooke Hospital in Cambridge for operations, one was sent to the workhouse being of unsound mind, one was discharged being an unfit patient, and one for insubordination, one left at her own request, and seven remained under treatment at the end of the year. The cost per bed has been reduced to £51 15s. 4d., but a heavy outlay for necessary alterations leaves the hospital with no larger balance than it had last year.

